# G-Bean: an ontology-graph based web tool for biomedical literature retrieval

**DOI:** 10.1186/1471-2105-15-S12-S1

**Published:** 2014-11-06

**Authors:** James Z Wang, Yuanyuan Zhang, Liang Dong, Lin Li, Pradip K Srimani, Philip S Yu

**Affiliations:** 1 School of Computing, Clemson University, Clemson, SC, 29634, USA; 2 Barnes and Noble, LLC, New York City, NY, 10007, USA; 3 Department of Computer Science & Software Engineering, College of Science and Engineering, Seattle University, Seattle, WA, 98122, USA; 4 Department of Computer Science, University of Illinois at Chicago, Chicago, IL, 60607, USA

## Abstract

**Background:**

Currently, most people use NCBI's PubMed to search the MEDLINE database, an important bibliographical information source for life science and biomedical information. However, PubMed has some drawbacks that make it difficult to find relevant publications pertaining to users' individual intentions, especially for non-expert users. To ameliorate the disadvantages of PubMed, we developed G-Bean, a **g**raph based **b**iomedical s**ea**rch e**n**gine, to search biomedical articles in MEDLINE database more efficiently.

**Methods:**

G-Bean addresses PubMed's limitations with three innovations: (1) *Parallel document index creation*: a multithreaded index creation strategy is employed to generate the document index for G-Bean in parallel; (2) *Ontology-graph based query expansion*: an ontology graph is constructed by merging four major UMLS (Version 2013AA) vocabularies, MeSH, SNOMEDCT, CSP and AOD, to cover all concepts in National Library of Medicine (NLM) database; a Personalized PageRank algorithm is used to compute concept relevance in this ontology graph and the Term Frequency - Inverse Document Frequency (*TF-IDF*) weighting scheme is used to re-rank the concepts. The top 500 ranked concepts are selected for expanding the initial query to retrieve more accurate and relevant information; (3) *Retrieval and re-ranking of documents based on user's search intention*: after the user selects any article from the existing search results, G-Bean analyzes user's selections to determine his/her true search intention and then uses more relevant and more specific terms to retrieve additional related articles. The new articles are presented to the user in the order of their relevance to the already selected articles.

**Results:**

Performance evaluation with 106 OHSUMED benchmark queries shows that G-Bean returns more relevant results than PubMed does when using these queries to search the MEDLINE database. PubMed could not even return any search result for some OHSUMED queries because it failed to form the appropriate Boolean query statement automatically from the natural language query strings. G-Bean is available at http://bioinformatics.clemson.edu/G-Bean/index.php.

**Conclusions:**

G-Bean addresses PubMed's limitations with ontology-graph based query expansion, automatic document indexing, and user search intention discovery. It shows significant advantages in finding relevant articles from the MEDLINE database to meet the information need of the user.

## Background

Currently, one of the most important bibliographical information sources for life science and biomedical research is MEDLINE database [1]. Building a Web-based tool to find relevant biomedical literature in MEDLINE database in response to a query remains a challenge due to the increase in volume and diversity of topics of biomedical literatures [2]. The primary portal to search the MEDLINE database is PubMed [3] by National Center for Biotechnology Information (NCBI). However, finding relevant publications with PubMed pertaining to users' individual interests is still daunting, especially for non-expert users. Due to the difficulty of forming appropriate query statements, only experienced users such as librarians [4] can obtain accurate search results using PubMed interface. It is widely reported that less-experienced users, including those who regularly use the PubMed system, do not utilize it as effectively as experienced users [5-7]. Those less-experienced users either fail to employ the most relevant context-sensitive keywords or fail to effectively formulate query expressions using Boolean logic [8,9]. It has been reported [8] that a novice user (e.g. a third year medical student) requires an average of fourteen separate queries to get the desired information.

In addition, PubMed does not always return the most relevant articles for user queries. PubMed's underperformance in biomedical information retrieval is partly due to the fact that it uses only a very small subset of the Medical Subject Headings (MeSH) [10] to index the biomedical articles. MeSH (ver. 2013) consists of 26,853 descriptors, 83 qualifiers, over 213K assisting entry terms, and over 214K supplementary concept records. However, PubMed uses only the descriptors and qualifiers for indexing purposes. It means only 5.9% of all concepts in MeSH are used for the document indexing. While there are 2.9 million biomedical concepts enumerated in UMLS Metathesaurus 2013AA [11], the PubMed index utilizes less than 1% of this available vocabulary. Many studies, such as Hersh [4,12,13], Yoo [14] and Abdou [15], have attempted to address PubMed's low vocabulary coverage problem by expanding user queries with more concepts in MeSH ontology. However, it has been observed that these query expansion approaches [14,15] offer no significant advantages over the free-text based search methods; missing concepts and incomplete synonym sets (due to the use of only MeSH ontology) were found to be the major causes of the inadequacy of existing query expansion schemes.

To address these issues, we develop a new Web-based literature retrieval tool, G-Bean (a **g**raph based **b**iomedical s**ea**rch e**n**gine), to query documents in MEDLINE database. The major contributions of G-Bean are three folds: 1) it uses a multithreaded parallel algorithm to automatically generate the document index to address the inefficiency of the PubMed's manual indexing process; this automated index generation scheme allows incremental index update for timely index maintenance; 2) it merges multiple biomedical ontologies into a single ontology graph and uses all concepts in this ontology graph to index documents, ameliorating PubMed's low concept coverage problem of using only MeSH terms for indexing; 3) it automatically retrieves additional relevant articles based on user's current selection and ranks them according to their semantic similarities with all articles selected so far while PubMed can recommend a list of articles matching the same keywords with only the current viewing article [16].

## Methods

### Ontology-graph construction

In G-Bean, we employ a subset of UMLS Metathesaurus as the knowledge source to construct an ontology graph. The Metathesaurus of UMLS is the largest vocabulary database that contains information about biomedical and health related concepts and inter-relationships among concepts [17]. Each biomedical concept in the Metathesaurus is a grouping of synonymous terms and is identified by a distinct eight character alphanumeric string, called Concept Unique Identifier (CUI). The CUI is linked to a set of lexical variants strings, which is an alternative way to represent the concept. The MRCONSO table contains information of these CUIs to resolve synonymy problems that may arise in organizing medical text. Information includes concept-names, spelling variations, and acronyms, etc. The inter-concept relationships, such as parent/child, and immediate siblings, are stored in the MRREL table. Our ontology graph is automatically constructed using the information from MRCONSO and MRREL tables where a vertex represents a concept and an edge represents an inter-concept relationship.

Since four ontologies in UMLS, MeSH, SNOMEDCT, CSP and AOD cover all senses of the target words in NLM database [18], we obtain these four ontologies from UMLS 2013AA [11] to construct the ontology graph with the assistance of UMLS MetamorphoSys, a multi-platform UMLS installation tool for UMLS Knowledge Sources [19].

### Parallel index creation for MEDLINE database

We first download the 2014 MEDLINE/PubMed Baseline database which contains 22,376K records from NLM [20] and then adapt Lucene Java search library (version 4.5.1) to create index for MEDLINE documents on the G-Bean server [21]. Each MEDLINE citation has a unique PubMed identifier called PMID. Since MEDLINE citation records do not contain full text articles, only the title and abstract of the documents are processed and indexed. A modified Lucene standard analyzer with an enhanced MIT stop-list and the Porter stemmer is used to analyze (process special characters), tokenize (break into words), stem (get base of word) and index MEDLINE document's title and abstract respectively. Moreover, MetaMap 2013 is employed to map the title and the abstract into a set of associated CUIs which are then indexed with our multithreaded indexing process.

The size of the 2014 MEDLINE/PubMed Baseline database is over 160GB. Building an index for this large dataset is challenging with Lucene library. It takes more than 10 days to generate the entire index for all MEDLINE documents using a computer with system parameters shown in Table 1.

**Table 1:** Physical configuration of the computer used to create index of MEDLINE documents

CPU	Intel Core i7-2600 CPU @ 3.40GHz
**The number of cores**	4

**Memory**	8GB

**System type**	X86_64

To speed up the index creation, we modify Lucene Java search library to make it multithread capable. We use a threadpool [22] to submit multiple tasks to the multi-core computer for MEDLINE index creation. The thread pool is created by using ThreadPoolExecutor, as shown below:

*new ThreadPoolExecutor(int corePoolSize*,

*int maximumPoolSize*,

*long keepAliveTime*,

*TimeUnit unit*,

*BlockingQueue<Runnable> workQueue*,

RejectedExecutionHandler handler)

where *corePoolSize *is the number of threads in the core pool, *maximumPoolSize *indicates the maximum number of threads allowed in the thread pool, *keepAliveTime *(i.e., thread keep-alive time) is the amount of time that waiting threads in excess of the core pool size may remain idle before being terminated, *unit *identifies the time unit for the *keepAliveTime *argument, *workQueue *is the queue to hold tasks before they are executed, *handler *demonstrates the handler that blocks the execution when maximum number of threads has been reached or queue capacities are exceeded. Since index creation is mostly a CPU-bound task as it does not involve much of I/O operations, the *corePoolSize *is set close to the number of CPU cores for our index creation.

The MEDLINE dataset contains 746 compressed files. During index creation, the RAM size required by Lucene is determined by the buffer size used by IndexWriter. To maximize the throughput of index creation, we should set the IndexWriter buffer as large as possible. Ideally, we should create the index for all 746 files at once. However, due to the memory space limitation in the computer we used, we divided the 746 files into 8 groups and created one index for each group. Then we use IndexWriter's addIndexes method to merge these 8 partial indices into the final index for the entire 746 files. With this approach, the total time for creating the MEDLINE index is:

(1) $$T_{p}=\overset{8}{\underset{i=1}{\sum }}T_{i}+T_{merge}$$

where *T_i _*is the time to create the partial index for files in group *i , T_merge _*is the time to merge the 8 partial indices into the final index.

### Evaluation of multi-thread based parallel index creation

To evaluate how the multithreaded parallel index creation approach speeds up the creation of MEDLINE index, we compare the total time for creating the MEDLINE index using this new approach with that using the original Lucene library on the same computer as shown in Table 1. Experiments show that the best performance is achieved using *ArrayBlockingQueue *with the following settings:

$$n_{cps}=4,{n_{mp}}_{s}=5,n_{queue}=18$$

where *n_cps _*is the value of *corePoolSize, n_mps _*is the value of *maximumPoolSize *and *n_queue _*is the size of *ArrayBlockingQueue*.

We run our experiment three times and obtain the average time to create the MEDLINE index using the multithreaded approach and the original Lucene library respectively. The average time for creating the MEDLINE index using our multithread approach is 250,917 seconds (less than 3 days), while the average time used for creating the MEDLINE index with the original Lucene library is 913,340 seconds (more than 10 days); the multithreaded index creation achieved a speed-up of 3.64 over the original Lucene library on the same computer with 4 CPU cores. The resulting index of the MEDLINE database occupies 30.4 GB disk space. Since our proposed method computes the indices for 8 groups of files separately and merge the partial indices into the final index, we can also update the index weekly through merging the index of the newly posted documents with the existing index. The newly posted documents can be downloaded from MEDLINE database [23].

### Ontology-graph based query expansion scheme

Query expansion is widely used to reconstruct a seed query by adding extra related words to the input query with the purpose of matching additional related documents. It is helpful to retrieve potential relevant documents not indicated by initial query [24-27]. After getting the input query from the user, G-Bean expands the query with additional related words retrieved from the constructed ontology graph. The query expansion process first uses MetaMap 2013 [28] to map the input query to concepts in UMLS to find the CUIs representing the input text. These mapped CUIs are marked as Original CUIs and are used as the initial teleportation probability vector for the Personalized PageRank algorithm, which is applied over the ontology graph to calculate the Personalized PageRank Values (PPV) for concepts in the ontology graph [29-31]. The PPV represents the relevance of a concept to the query. We call these CUIs labeled with their PPVs as PPV CUIs. The top 500 ranked PPV CUIs are selected as the concept candidates for query expansion.

Because the original CUIs are used as the initial teleportation probability vector in PPV computation, these CUIs will always have high PPV scores and will always be ranked at the top of the PPV CUI list. Thus, the direct use of PPV CUIs for query expansion does not make any significant difference from simply using the Original CUIs. Furthermore, Personalized PageRank algorithm has a tendency of ranking the general concepts (frequently occurred) higher than more specific concepts since there are more links to general concepts. Thus, simply selecting the top concept candidates from the PPV CUI list for query expansion might greatly decrease the query accuracy since more general terms are included in the expanded query. To alleviate this problem, we employ a *TF-IDF *weighting scheme to re-rank the PPV CUIs. The OHSUMED documents, a clinically-oriented MEDLINE subset which consists of 348,566 documents covering references from 270 medical journals, are used to build the *IDF *repository to estimate the popularity of the PPV CUI in all OHSUMED documents. We calculate a weight value for the concept ranked #*i *among the PPV CUI list using formulas shown in (2) and (3):

(2) $$idf_{i}=\text{max}\left\{0,\text{log}\left(\frac{N-n_{i}+0.5}{n_{i}+0.5}\right)\right\}$$

(3) $$w_{i}=p_{i}^{\gamma }\cdot idf_{i}$$

where *w_i _*is the weight, *p_i _*is the *L_1_*-normalized PPV score for this concept (the calculation of PPV score for concept is discussed in [32]), *γ*∈[0,1] is a tuning parameter used to increase the $p_{i}^{\gamma }$ by decreasing *γ, idf_i _*represents the inverse document frequency of this concept, *N *is the total number of documents in *IDF *repository, and *n_i _*specifies the number of documents in *IDF *repository that contain this concept.

The PPV CUIs are re-ranked by their weights and top ranked ones are used to expand the query. Extensive experimental results show that the query expansion scheme of G-Bean outperforms the popular Lucene approach by 22% while other existing query expansion approaches are unable to beat the free-text based Lucene strategy [32].

### Document retrieval

When a query is entered, query text is analyzed by the same Lucene analyzer used to create the index, with an enhanced MIT stop-list and the Porter stemmer to extract query terms. MetaMap 2013 is used to map the query text into Metathesaurus CUIs. The query is expanded with the ontology-graph based query expansion scheme discussed in the previous section and the expanded query is submitted to G-Bean for searching the relevant documents.

**Figure 1:**
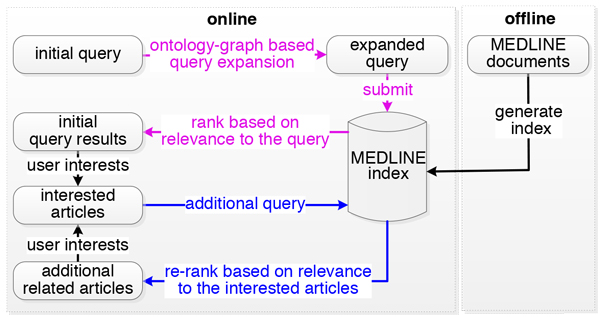
**Major steps of search process in G-Bean**.

After the user reviews a returned article, he/she can indicate if he/she is interested in the article. G-Bean can form a new query using the key concepts automatically obtained from all articles that are interested by the user and retrieve a list of new articles that are relevant to all articles selected by the user. Therefore, the user does not need to browse through the long list of initial search results to find new articles related to their interested articles. PubMed can also display a list of recommended articles when a user is viewing one particular article. However, PubMed can recommend the articles only related to the current viewing article based on keywords matching. Thus, the recommendation may not accurately reflect the user's true search intention. In addition, keywords matching may return inaccurate results due to polysemy problem of natural language; it may miss some relevant articles due to the synonymy problem of natural language. G-Bean utilizes the ontology-graph based query expansion to minimize these problems. With more articles selected, G-Bean can accurately determine the user's true search intention by analyzing the articles that the user is interested in and provides better recommendation, especially for interdisciplinary research articles. The major steps of a G-Bean search are shown in Figure 1.

### G-Bean implementation

**Figure 2:**
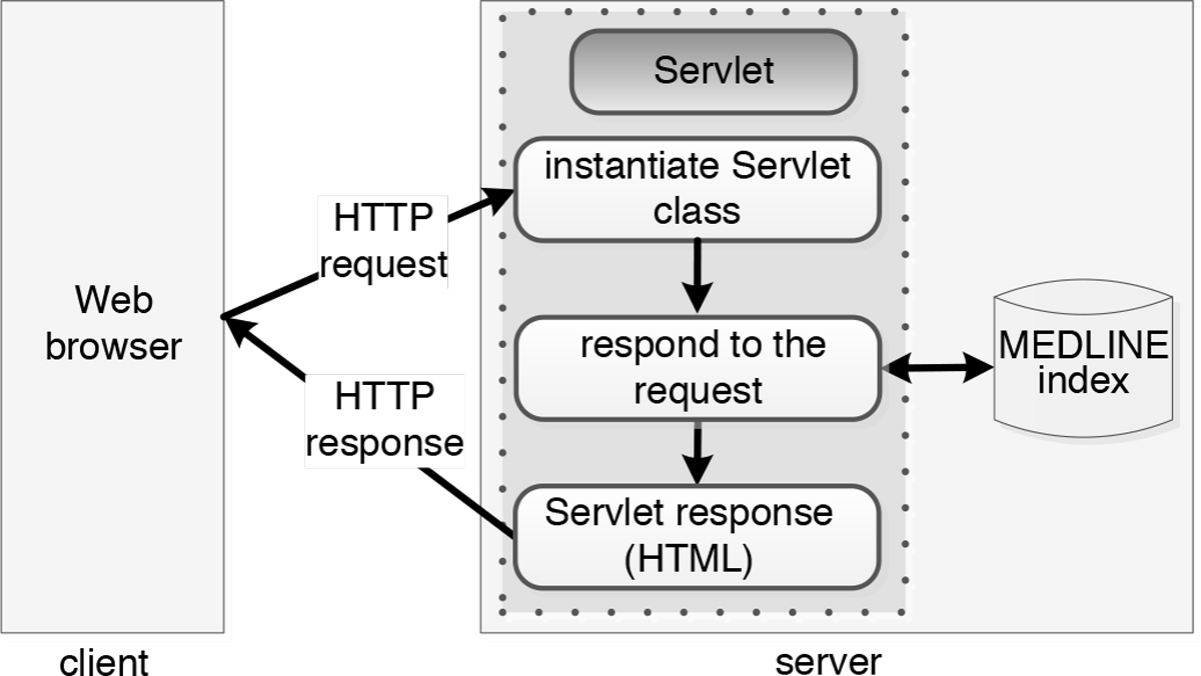
**The architecture of G-Bean**.

We have implemented and published G-Bean as a Web-based application which accepts any biomedical related user query and returns related articles in MEDLINE database. We use the Client-Server architecture powered by Java Servlet Pages (JSP) to implement the G-Bean system since the Java version of Lucene library was used to index the MEDLINE documents. The communication between the client and the server follows the HyperText Transfer Protocol (HTTP). The architecture of G-Bean consists of client-side and server-side components, as shown in Figure 2.

#### Client-side implementation

**Figure 3:**
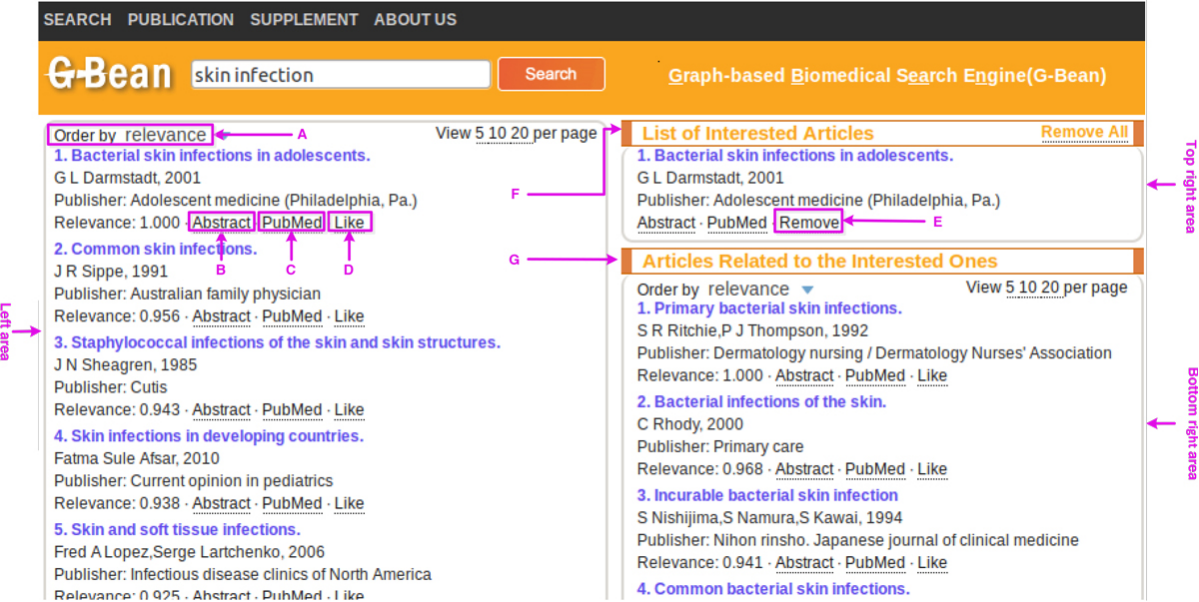
**Screenshot of the user interface of G-Bean**.

A G-Bean client is developed as a Web application. The JSP script collects the query from the user, dispatches it to the server and displays the search results. The user interface of G-Bean is illustrated in Figure 3. Search results are presented in three areas under the search bar: the left area lists the articles returned by user's initial query; the top right area lists the articles which the user is interested in and the bottom right area lists the articles related to all articles the user is interested in.

**A**synchronous **J**avaScript **A**nd **X**ML (Ajax) technique is employed to implement the following functions due to its ability to make partial page updates without reloading the whole page. *1) Document retrieval: *G-Bean provides an easy-to-use interface for user to retrieve documents related to the query from the MEDLINE database. Clicking the "Search" button triggers the server to retrieve articles related to the input query; *2) Document ranking: *the returned articles in the left area are ranked by relevance to the query by default. G-Bean allows the users to rank the returned articles by date, author name and title as well. As shown in Figure 3, the user can select the ranking criterion in the drop-down list A to rank the returned articles according to his/her need; *3) Document preview: *for a particular article, clicking the "Abstract" link (B in Figure 3) allows the user to preview the abstract; *4) PubMed article retrieval: *G-Bean provides the PubMed link for each returned article, which allows the user to retrieve information about the article in PubMed. Clicking the "PubMed" link (C in Figure 3) opens a new window to display the PubMed record for this article; *5) User intention discovery: *G-Bean allows the user to select articles of interest so that it can capture the user's search intention and to retrieve a list of articles relevant to all interested articles. If the user is interested in a returned article after viewing it, he/she can click the "Like" link (D in Figure 3) to add this article to the article list of interest (F in Figure 3); if a user changes his/her mind and wants to remove an article from the list F, he/she can click the "Remove" link (E in Figure 3); *6) Additional related articles update: *as long as the articles in list F are updated, the server retrieves articles related to those articles. The newly retrieved articles are presented in the bottom right area (G in Figure 3). These articles are ranked by the relevance to all articles in list F by default. Users can select to rank the newly retrieved articles by date, author name and title as well.

#### Server-side implementation

**Figure 4:**
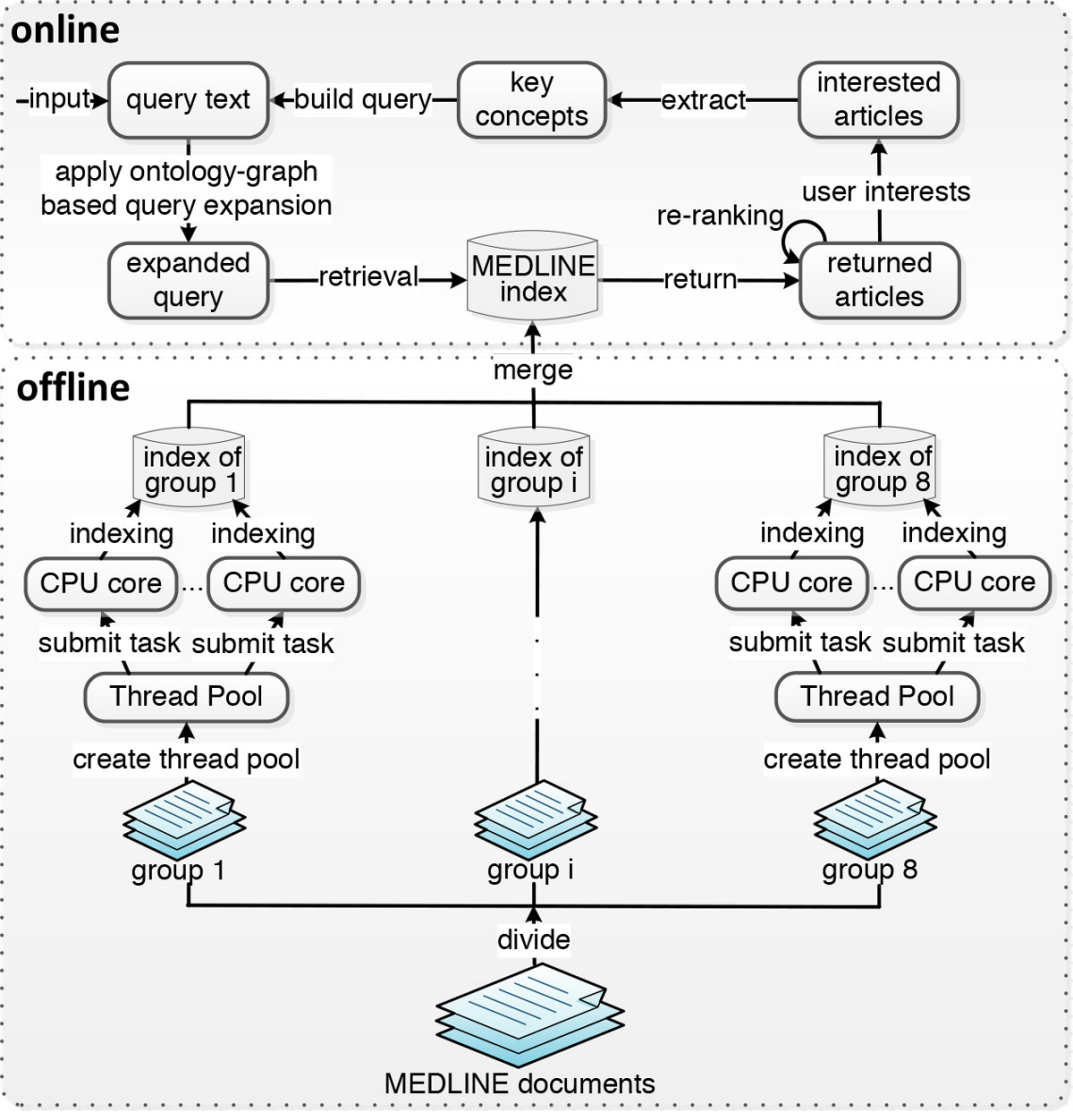
**Major components of the G-Bean server**.

G-Bean uses Server Applet (Servlet) to receive and respond to requests from clients via HTTP; Apache Tomcat is used as the Servlet container to manage the Servlet. The data flow at the server-side is shown in Figure 4. The document index is created offline by our proposed multi-thread process. The online part of the server side functions includes query expansion, key concept extraction, and search results ranking.

## Results

To evaluate G-Bean's search performance, we conducted a subjective evaluation using the 106 benchmark queries from the OHSUMED dataset, which is generated by clinicians in course of their patient care. The 106 queries consist of patient information (a brief statement about the patient) and information need (a clinician's information request statement for the patient) fields [33].

We invited 20 graduate students in Clemson University to use these 106 benchmark queries to search the MEDLINE citations through G-Bean and PubMed respectively. The returned results on both search engines are set to be ranked by their relevance to the query. The students carefully examined the results returned by both search engines for each query, and decided independently which search engine produced more relevant search results. For a given query, they were asked to choose one of the following three answers after carefully reviewing the search results:

(a) G-Bean returns better search results than PubMed does;

(b) PubMed returns better search results than G-Bean does;

(c) G-Bean and PubMed return similar search results.

After collecting all answers from the students, we summarize the subjective search performance comparison between G-Bean and PubMed for each query into 5 categories:

• G-Bean and PubMed return similar search results;

• G-Bean is definitely better than PubMed;

• G-Bean is marginally better than PubMed;

• PubMed is definitely better than G-Bean;

• PubMed is marginally better than G-Bean.

Given a query, let *n_a_, n_b_, n_c _*denote the number of students who chose (a), (b), and (c) respectively. We consider G-Bean and PubMed return similar search results iff *n_c _*≥ 10 or *n_a _= n_b _*. Otherwise, we deem:

• G-Bean is definitely better than PubMed iff *n_b _= *0 or (*n_a _− n_b _*)/*n_b _*≥ 25%;

• G-Bean is marginally better than PubMed iff *n_b _>*0 and 0 < (*n_a _− n_b_*)/*n_b _*< 25%;

• PubMed is definitely better than G-Bean iff *n_a _*= 0 or (*n_b _− n_a _*)/*n_a _*≥ 25%;

• PubMed is marginally better than G-Bean iff *n_a _>*0 and 0 < (*n_b _− n_a _*)/*n_a _*< 25%.

Our summary, as shown in Table 2, indicates that G-Bean returned definitely better search results in 67 of these benchmark queries and marginally better search results in 12 of these benchmark queries, while PubMed retuned definitely better results in only 7 of these queries and marginally better results in 1 of these queries. Overall, G-Bean returned better search results in 79 of these benchmark queries while PubMed returned better search results in only 8 of these benchmark queries. For the remaining 19 queries, these two search engines returned similar search results. This subjective evaluation confirms the efficiency of G-Bean search engine on biomedical information retrieval. The details of the subjective evaluation experiment are available at http://bioir.cs.clemson.edu:8080/BioIRWeb/supplement.jsp.

**Table 2:** Performance comparison between G-Bean and PubMed using the OHSUMED 106 queries

Number of queries that G-Bean returned definitely better results	67
Number of queries that G-Bean returned marginally better results	12

Number of queries that PubMed returned definitely better results	7

Number of queries that PubMed returned marginally better results	1

Number of queries that the two search engines returned similar results	19

It is worth-noting that no student could find any relevant article using PubMed for some queries such as queries #17, #52, and #95. For some other queries, such as queries #23, #49, #71 and #89, PubMed only returned one result in each case. Pursuing further investigation, we found that PubMed assumed "AND" operators for keywords in a query string. For instance, in query #17: "Rh isoimmunization, review topics", PubMed obtained four keywords, *Rh, isoimmunization, review*, and *topics*. It assumed "AND" operation on these keywords to form the query for searching the MEDLINE database, i.e., it tried to retrieve articles containing all these four keywords. As a result, PubMed returned no result for OHSUMED query #17 as shown in Table 3 because there is no article in MEDLINE database containing all these four keywords. Unfortunately, none of the graduate students found this problem in their evaluation of PubMed search interface, nor did they figure out how to get a better search result using PubMed. Actually, if we take out the keyword "topics" from the query #17 and submit it to PubMed, it returns relevant articles as shown in Table 4. For an experienced user, it is not very hard to form a proper query string (with some Boolean operators) to search the intended articles. However, for a novice user, such as a graduate student, it is frustrating when the query returns no search results. In an extreme situation, if a user happens to input a keyword not in any of the articles, e.g., a typo, no result will be returned. In addition, PubMed uses MeSH to index documents. If a query contains no MeSH terms, PubMed may either return no search results or return irrelevant results after a long period of search. On the other hand, as shown in Table 3, G-Bean returned articles closely related to the query in most of the cases. We note that these search results are obtained in January, 2014. Since MEDLINE database updates periodically, search results obtained later may be different from those reported in this paper.

**Table 3:** Top 5 returned results for query #17 "Rh isoimmunization, review topics" in G-Bean and PubMed

Rank	G-Bean		PubMed	
	**Title**	**PMID**	**Title**	**PMID**

**1**	A review of 58 Rh-isoimmunized cases.	3021092	No item returned.	

**2**	Suppression of Rh isoimmunization. A review.	101918		

**3**	Isoimmunization in pregnancy.	15145363		

**4**	Rhesus isoimmunization in twin gestation.	6433714		

**5**	Isoimmunization with anti-U antibody.	6716502		

**Table 4:** Top 5 returned results for query "Rh isoimmunisation, review" in PubMed

Rank	PubMed	
	**Title**	**PMID**

**1**	Postpartum Rh immunoprophylaxis	23168770

**2**	Performance in appropriate Rh testing and treatment with Rh immunoglobulin in the emergency department	22153971

**3**	Diagnostic laboratory technologies for the fetus and neonate with isoimmunization	21641488

**4**	Management and prevention of red cell alloimmunization in pregnancy: a systematic review	23090532

**5**	Prediction of the rate of decline in fetal hemoglobin levels between first and second transfusions in red cell alloimmune disease	22949399

## Conclusions

In this paper, we develop G-Bean for biomedical information retrieval from MEDLINE database. Four major ontologies, MeSH, SNOMEDCT, CSP and AOD, which cover all concepts in NLM database, are used to build an ontology graph. An ontology graph based query expansion scheme is used to expand the input query with additional more specific query terms to retrieve relevant articles more accurately. To address the weakness of manual indexing mechanism used in PubMed, we use a multithreaded parallel program to speed up the index creation so that we can timely update the document index for information retrieval. By discovering the user's true search intention based on articles he/she is interested in, G-Bean shows significant advantage in retrieving relevant articles compared to PubMed's search interface. Our performance study shows that search results returned by G-Bean are more relevant than those returned by PubMed using the 106 benchmark queries from OHSUMED dataset.

## List of abbreviations used

Ajax: **A**synchronous **J**avaScript **A**nd **X**ML; CUI: **C**oncept **U**nique **I**dentifier; G-Bean: **G**raph based **B**iomedical S**ea**rch E**n**gine; HTTP: **H**yper**T**ext **T**ransfer **P**rotocol; JSP: **J**ava **S**ervlet **P**ages; MeSH: **Me**dical **S**ubject **H**eadings; NCBI: **N**ational **C**enter for **B**iotechnology **I**nformation; NLM: **N**ational **L**ibrary of **M**edicine; PMID: **P**ub**M**ed **Id**entifier; PPV: **P**ersonalized **P**ageRank **V**alue; Servlet: **Serv**er App**let**; TF-IDF: **T**erm **F**requency - **I**nverse **D**ocument **F**requency.

## Competing interests

We declare having no competing interest.

## Authors' contributions

JZW participated in the design of the study and helped to modify the manuscript. LD developed the ontology-graph based query expansion, and participated in drafting the manuscript and designing the G-Bean website. YZ carried out the evaluation experiment, implemented the G-Bean website, created the MEDLINE index and participated in drafting the manuscript. LL participated in designing the G-Bean website. PKS participated in the design of the study and helped to modify the manuscript. PSY reviewed the draft and provided feedbacks in the direction of the research and final manuscript. All authors read and approved the final manuscript.

## Declarations

Publication of this article has been funded by National Science Foundation [DBI-0960586 and DBI-0960443]; National Institute of Health [1 R15 CA131808-01 and 1 R01 HD069374-01 A1]; Guangzhou Science and Technology Fund of China [2012J4300038, LCY201206, 2013J4300061]; Guangdong Science and Technology Fund of China [2012B091100221].

This article has been published as part of *BMC Bioinformatics *Volume 15 Supplement 12, 2014: Selected articles from the IEEE International Conference on Bioinformatics and Biomedicine (BIBM 2013): Bioinformatics. The full contents of the supplement are available online at http://www.biomedcentral.com/bmcbioinformatics/supplements/15/S12.
